# Convergence between Cardiometabolic and Infectious Diseases in Adults from a Syndemic Perspective: A Scoping Review

**DOI:** 10.3390/tropicalmed9090196

**Published:** 2024-08-26

**Authors:** Silvia Quiroz-Mena, Juan Gabriel Piñeros-Jimenez, Wilson Cañon-Montañez

**Affiliations:** 1 Faculty of Health, Technological University of Choco “Diego Luis Cordoba”, A.A 292, cra 22 No.18B-10, Quibdó 270001, Colombia; 2 National Faculty of Public Health, University of Antioquia, Medellin 050010, Colombia; juan.pineros@udea.edu.co; 3 Faculty of Nursing, University of Antioquia, Medellin 050010, Colombia; wilson.canon@udea.edu.co

**Keywords:** syndemics, cardiovascular diseases, communicable diseases, public health

## Abstract

**Objective**. Synthesize the approaches used to study the convergence between cardiometabolic and infectious diseases in adults from a syndemic perspective based on the scientific evidence available to date worldwide. **Methods**. Scoping review that follows the recommendations of the PRISMA statement. The protocol was registered in INPLASY202150048. The search for studies was carried out in MEDLINE, LILACS, Web of Science and Scopus. **Results**. Since the COVID-19 pandemic, there has been an increase in studies in the field of convergence between cardiometabolic and infectious diseases from a syndemic perspective, but only three studies were classified as true syndemics. There are weaknesses in the adherence to the elements of the syndemic theory, given a low incorporation of population measurements, and until now it has not been possible to find convincing empirical evidence that supports the bio–bio interface. Quantitative methods predominated through models focused on “sum scores”. **Conclusions**. Future studies should comprehensively address the elements of syndemics, review discrepancies between additive analyses versus other modeling, and incorporate the influence of large-scale social forces. The lack of these aspects distances studies from the notion of syndemic, bringing them closer to comorbidity or multimorbidity approaches.

## 1. Introduction

The convergence between cardiometabolic and infectious diseases is not a new issue [1]. Since the 1970s, there have been reports of studies, the results of which show unidirectional relationships, where the infectious agent is an exposure factor that increases the risk or promotes the development of cardiometabolic disease, such as periodontal infection and atherosclerosis [2]. On the other hand, in bidirectional relationships, both diseases can assume the role of exposure and effect. Diabetes mellitus (DM) can lead to a more severe form of tuberculosis (TB) and affect its management, while TB causes impaired glucose tolerance and makes glycemic control difficult in patients with DM [3].

The above is summarized in a pathogen–pathogen view, which has been expanded when trying to understand, where, under what conditions, in whom and how. These convergences occur [1] and add another layer of complexity, going beyond traditional notions of comorbidity and multimorbidity [4]. Syndemic theory, described by medical anthropologist Merrill Singer, offers an opportunity to understand how certain diseases grouped in various social contexts and within specific populations. This theory involves the assessment of two key elements: (i) concentration—grouping of two or more diseases due to the influence of large-scale demographic, social, economic and political factors; and (ii) interaction—these diseases interact through various biological mechanisms (interface bio–bio, such as inflammation) and/or social (bio–social interface, such as stigma). As a result of their interaction, greater adverse health outcomes are generated (excess disease burden and mortality) [5].

One of the challenges has been to achieve coherence between what is stipulated in the theory and the conceptual and methodological approaches used in the analyses [6]. A review of the trends in syndemic research between 2015 and 2019 showed that only 12% of the articles evaluated respond appropriately to the definition of syndemic [7].

There are some reviews on the topic, but these do not focus on the convergence between cardiometabolic and infectious diseases; they do not delve into the types of convergences, methods used and their coherence with theory. In addition, previous reviews include studies before the COVID-19 pandemic, when the rise of the term syndemic became more relevant [7,8]. The appropriation of the elements of syndemic theory must be examined and standardized to guarantee valid results and enhance its usefulness in clinical and public health practice in the face of future pandemics.

The objective of this review is to synthesize the approaches used to study the convergence between cardiometabolic and infectious diseases in adults from a syndemic approach based on the available scientific evidence.

## 2. Materials and Methods

A scoping review was carried out under the standards established in “PRISMA extension for scoping reviews (PRISMA-ScR)” [9]. The protocol was registered in INPLASY202150048 [10].

Literature searches were carried out in four databases (from inception to October 2023). A combination of English descriptors in MEDLINE, LILACS, Web of Science and Scopus was used using the following search equation: (syndemic OR syndemics) AND (cardiometabolic OR metabolic OR cardiovascular OR noncommunicable diseases OR noncommunicable diseases OR non-infectious Diseases) AND (communicable diseases OR Infectious diseases OR infections OR infection) (Tables S1 and S2).

No language or year of publication restrictions were established. A filter was applied by age (studies over 18 years of age), and in the title and abstract review stage, the following exclusion criteria were used:

- They do not include the two diseases of interest (cardiometabolic and infectious).
- They do not involve convergence between the two types of diseases.
- They are review studies, editorials, case reports, comments or letters to the editor.

From this initial screening process, the studies were selected for full-text review. Subsequently, those studies that did not explicitly refer to a syndemic approach in any section of the article were excluded. The reasons for the non-inclusion of certain documents were listed and discussed by the researchers.

For the extraction and management of data from the definitive studies, a database was created in Microsoft Excel version 16 with the following information: bibliographic database, authors, title, year, country, participants, diseases or syndemic conditions, research design, methods and adverse outcomes.

The data synthesis strategy was qualitative, given the objective of the review, which seeks to describe the different approaches used to evaluate syndemics. For this, the instrument “JBI QARI Data Extraction Form for Interpretive and Critical Research” was used [11]. The synthesis was developed in three steps: (1) extraction of all the findings included in the selected documents; (2) grouping the results into similar themes; and (3) unifying the results into previously defined categories of interest according to the key elements associated with the syndemic theory and in consensus with the research team.

The methodological quality of the studies was assessed using a tool for assessing the quality of diverse studies (QuADS), which allows the assessment of methodological and reporting quality in systematic reviews of mixed or multiple methods studies [12]. Additionally, the “robvis” tool was used to visualize the evaluation of the quality of the studies and the risk of bias [13].

## 3. Results

**Figure 1:**
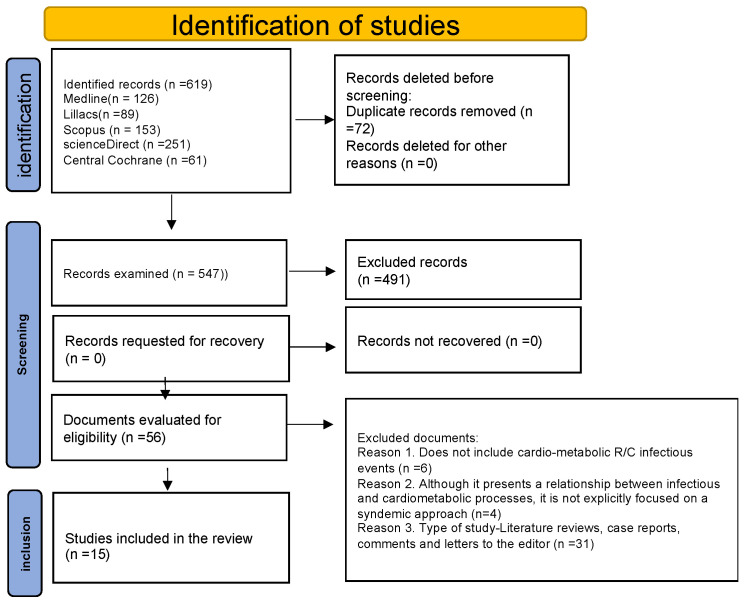
Flowchart—PRISMA [9].

The database searches identified 619 publications, of which 56 articles were chosen for full-text selection after reading titles and abstracts. Of this total, 41 did not meet the inclusion criteria, leaving 15 eligible articles. The selection process is summarized in the PRISMA flowchart (Figure 1).

The selected studies were published between 2015 and 2023 [14,15,16,17,18,19,20,21,22,23,24,25,26,27,28]. The largest number of articles were published following the COVID-19 pandemic (n = 12), and were carried out in the United States (n = 7) and African countries (n = 3). Most of them are quantitative (n = 11) and cross-sectional (n = 12) studies. The number of participants varies in a range from 27 to 12,052 people with specific characteristics defined by age, sex, sexual orientation, ethnic/racial condition, geographic area, socioeconomic status, and index or underlying disease (Table 1). 

The syndemic conditions evaluated in the studies incorporate a count of the presence of two or more diseases or health conditions (HIV infection, hypertension, Diabetes mellitus, obesity, depression or anxiety), risk factors (stress or psychoactive substance consumption) and social determinants (low educational level, unemployment, violence, sexual abuse and child abuse). Some authors called the sum of these factors the syndemic burden. The main outcomes reported are linked to impairments in the progression, control and management of an index disease; lower quality of life; higher risks of complications; and death (Table 1).

**Figure 2:**
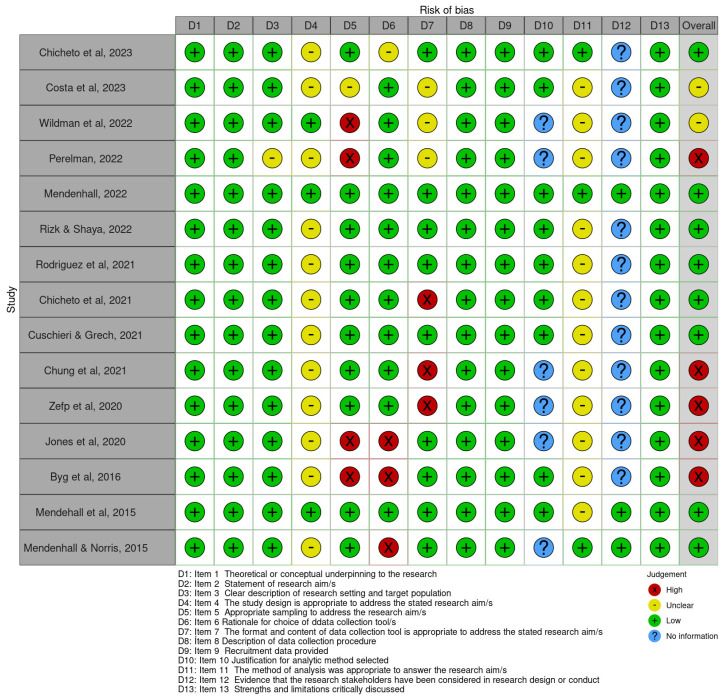
Results of the application of the quality assessment tool for studies with diverse methods (Quality assessment with diverse studies—QuADS).

The quality assessment, considering the QuADS tool, showed that all studies are based on a theoretical framework (syndemic theory); moreover, it also declared the objectives, described the collection procedures and provided recruitment data, but only 53% of the studies evaluated had a high methodological quality (n = 8). Problems related to the coherence between the methodological framework and the objectives were evident, as well as the lack of evidence on the participation of research stakeholders in its design and execution (Figure 2).

### 3.1. Level of Measurement and Application of the Key Elements of Syndemic Theory

Most studies focus on the individual level (n = 9). The element of concentration, defined as the coexistence of two or more diseases in defined temporal and geographic contexts due to detrimental social conditions, was evaluated in only eight studies. Bio–social interaction is the most evaluated element (n = 13), while the bio–bio interface is only addressed in four studies (Table 2).

Considering what was stipulated by Singer, Bulled and Ostrach in their text “Whither syndemics?: Trends in syndemics research, a review 2015–2019”, two initial categories were defined to classify the evaluated studies. Firstly, those studies that included both the population and individual level and the two key elements of the theory were classified as “syndemics”, that is, they analyze the spatiotemporal grouping and the true interactions of the evaluated cardiometabolic and infectious diseases or the health burdens within a specific population. Furthermore, they incorporate how the bio–bio interactions of these health burdens result in worse diseases, and shared social or structural factors within populations further exacerbate biological conditions creating true synergy rather than simply co-occurrence of disease Within this category, only three studies were included (Table 2).

Secondly, those studies that only focused on the individual level and did not fully develop the key elements of the theory, especially the bio–bio interaction or the mechanisms by which the cardiometabolic and infectious diseases are evaluated, were classified as “potential syndemics”. And social conditions interacted to exacerbate harmful health outcomes (n = 11) (Table 2).

### 3.2. Consistencies between the Underlying Conceptual Model, the Methodologies Used and the Postulates of the Syndemic Theory

Two conceptual models were represented based on the objectives set and the arguments described in the different studies. The first model presented groups-3 studies and attempts to represent in a unified manner approaches focused on evaluating the syndemic as the effect of the sum of a series of conditions related to cardiometabolic and infectious diseases on negative health outcomes, such as increased risk, complications and death in population groups with specific characteristics and/or a defined index disease.

**Figure 3:**
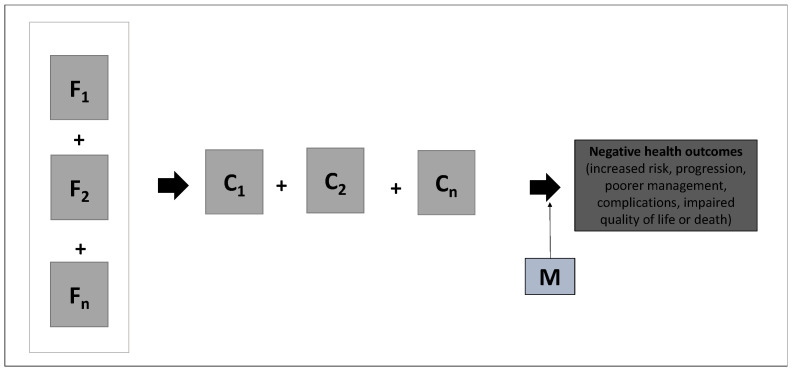
Conceptual model for addressing syndemics between cardiometabolic and infectious diseases according to the reviewed studies. F: Effect of social factors. C: Conditions (diseases and risk factors). M: Mediating factors (physiological or mental).

The second conceptual model presented groups-8 studies and involves not only the aspects described in the first model, but also incorporates the influence of social factors, reinforcing the bio–social interface. This model also incorporates the understanding of biological mechanisms related to the bio–bio interface (Figure 3). It should be noted that in the quantitative studies evaluated, the independent effect of each social factor included is measured.

The conceptual models represented incorporate a variety of both quantitative and qualitative methodologies. It is highlighted that the element of disease concentration was evaluated mainly with traditional epidemiological methods, specifically, estimation of measures of occurrence of the diseases and conditions of interest. These tend to be stratified by demographic and socioeconomic variables.

The interaction element was evaluated using bivariate and multivariate inferential techniques, including interaction terms in some cases. A dominant modeling focused on “sum scores” is evident. Qualitative studies address the elements of the theory through in-depth interviews, ethnographies and life stories. Mixed studies, which incorporate the variety of techniques described, were evident.

## 4. Discussion

This scoping review synthesized the approaches used to study the convergence between cardiometabolic and infectious diseases in adults from a syndemic perspective for the scientific evidence available to date. The COVID-19 pandemic showed complex and unexpected interactions among the diseases evaluated here, environmental factors and socioeconomic disparities, which makes the use of the syndemic theory important [16]. The results of this review become an opportunity to better understand this problem in order to address current and future pandemics.

The first finding of this review indicates that most studies focus only on the individual level. Strictly speaking, this finding is worrying, because if the origin of the term “syndemic” is reviewed, it is necessary to include the population level, for the term is derived from the Greek word “synergos”, which means two or more agents working together to create an effect greater than the sum of each one working alone. And we deduce that it refers to population, as it has been used in basic epidemiological concepts: epidemic, pandemic and endemic [5].

Additionally, falling into the individualistic fallacy, as proposed by Tsai, leads the syndemic area to lose opportunities to incorporate multilevel approaches, which allow us to understand how epidemics interact both at the level of populations and individuals, using as many ecological study designs as possible: cohort, case-control and cross-sectional studies. This author has incorporated important reflections on this aspect and has recently criticized how studies that ignore interactions with ecological influences to focus exclusively on the interaction of factors at the individual level can reach erroneous conclusions [29].

Other authors highlight that empirical evidence on syndemics require a greater understanding of the ways in which health conditions and social adversity interact in marginalized populations to further weaken these groups [30]. Emily Mendenhall, one of the great proponents of the theory, mentioned that “The COVID-19 syndemic is not global: context matters” because what drives the virus to move and interact with biological and social factors differs among countries and regions [31].

The second finding was linked to low adherence to the elements of the theory. Only three studies were classified as true syndemics since they incorporated both the individual and population levels and addressed aspects related to the concentration and interaction of diseases [15,18,27]. This has been reported in studies on the use of this theory before 2019 [7,8]. It has been mentioned that the confusion surrounding the non-application of these elements could be due to the fact that researchers are referring to conceptualizations from Singer’s early work. For this reason, we insist on the relevance of recognizing the developments that have been made over the years to articulate the elements in a complete and adequate manner [32].

So far, the empirical evidence supporting the bio–bio interface proposed in the theory is insufficient. Other systematic reviews have shown that most studies, especially epidemiological ones, have attempted to document the existence of syndemics only using the “sum score” specification, and it constitutes the dominant modeling in quantitative studies [29,33,34].

Since 1979, the concept of “interaction” has had a long and controversial history in the epidemiological literature. Renowned authors such as Rothman [35] and VanderWeele [36] have made contributions to direct the analysis of this element, providing foundations for its theoretical distinction and the implementation of formal tests to analyze the interaction within statistical models, ranging from interaction in additive models to the introduction of multiplicative terms [6].

The third finding shows that there is no single conceptual model to represent the syndemic between cardiometabolic and infectious diseases and that it depends on the nature of the interactions that are proposed. However, the studies evaluated have common characteristics in terms of their objectives and approaches, which allowed them to be grouped into two large types of conceptual models. The resulting models do not differ substantially from others proposed in previous literature reviews for other types of syndemics, where the interaction of factors, diseases and conditions in specific population groups is linked. For example, Tsai and Burns [37] in 2015 conducted a systematic literature review on syndemics of psychosocial problems and HIV risk, and as a result of the study, they constructed simple syndemic models in which two conditions (depression and substance abuse) coexist, and they are determined by the poverty and interact synergistically to increase HIV risk [37].

The fourth finding indicates a marked predominance of the positivist paradigm and the use of quantitative methods to address the syndemic between cardiometabolic and infectious diseases. However, it recognizes a strong incursion of qualitative methods, which are part of the anthropological roots of the theory and have been widely applied with other types of syndemics, especially those related to HIV and risk behaviors. The inclusion of mixed methods and longitudinal studies is highlighted as a response to the challenges posed in previous literature reviews [7,8]. In this order, expanding the spectrum of methods and understanding the type of syndemic addressed here has become an urgent need among researchers in this field.

Consistent with the results of this review, Tsai [29] indicates that future studies should aim to gain ground in the validation of the theory, and therefore, six aspects are suggested: (i) the clear exposure of the possible discrepancies that may arise among the additive analyses (score sum) versus those that test synergistic interactions from another type of modeling; (ii) the need to understand the complexity of large-scale social forces or structural determinants of health that influence the convergence of multiple diseases results; (iii) the use and triangulation of data from multiple sources; (iv) the assessment of the temporal effect of health risks following approaches such as the life course; (v) the comprehensive understanding of the effect of clinical and public health interventions for the management of these diseases; and (vi) the incorporation of studies at the population level and contextual effects, given that syndemics have been explicitly theorized as multilevel phenomena, as Singer highlights “the interaction between diseases occurs at both the population and individual levels” [6].

Not explicitly modeling social forces distances the field from political, economic and cultural explanations; downplays the first element of the theory (concentration); and brings it closer to notions of comorbidity or multimorbidity.

Finally, identifying and addressing syndemics globally contributes to the fulfilment of the United Nations 2030 Agenda for Sustainable Development Goals (SDGs) by adopting an integrative, collaborative and multi-sectoral perspective, which is in line with current approaches such as “One Health”. Both approaches are based on the recognition that health problems are often interconnected and cannot be addressed in isolation [38]. By considering the broader social, environmental and animal health contexts, the One Health approach can help identify and address the underlying factors that contribute to syndemics.

### Limitations

The results of this review must be interpreted with caution due to some limitations. First, the search strategy was restricted to studies reported in the English language, which could have overlooked other aspects reported in other studies in a non-English language. Second, it is likely that our review will have missed relevant articles that do not mention another type of convergence in their title, abstract or key words. On the same hand, we may not have captured all conceptual linkages with regard to common definitions within syndemics.

## 5. Conclusions

Syndemics appear to have played an important role in human history and are likely to continue to influence the global burden of disease today [5]. Understanding the syndemic between cardiometabolic and infectious diseases is an urgent issue, not only for facing the great challenges raised by COVID-19, but also for being able to cope with future pandemics and the emerging and re-emerging of public health threats.

With this scoping review, it would be expected that future empirical studies on syndemics would include both the individual and population levels within their approaches and would be natural candidates for the application of multilevel models. The findings of this review show the lack of adherence to the key elements of the syndemic theory. Therefore, the need also arises for future research to appropriate and incorporate the concentration of diseases and interaction as foundations when it comes to evaluating syndemics.

Linked to the above, it is necessary to include methodologies consistent with the elements of the theory. A recommendation that comes from this review and other studies is the use of mixed methodologies, the combination of different sources of information and exploration of data analysis methods. Additionally, the use of longitudinal studies that can more fully capture the complex interconnections among syndemic conditions is highlighted.

It is recommended that future studies achieve greater coherence among the objective, the theoretical framework (syndemics) and the methodology. This aspect is crucial to ensure logical research that increases the validity of the findings and reduces bias.

With respect to health interventions, multicomponent or comprehensive ones are suggested, yet more evidence is required, one that documents multiple pathways and encourages their use through advocacy for program implementers or policymakers. The study of syndemics involves drawing on various disciplines, including epidemiology and anthropology. This often means collaboration among researchers with different academic and professional backgrounds from different areas.

Clinical care and public health must consider the complexity of syndemics. They cannot be limited only to the understanding of comorbidities and the clinical management of convergent diseases [30]. Mastering the interaction of multiple diseases requires a change in interventions, to adequately manage not only infectious diseases but also the increasing burden of cardiometabolic diseases through a “one health” approach [2].

## Figures and Tables

**Table 1:** Characteristics of the included studies.

Author(s), Year	Country	Study Design	Participant Characteristics (n = Sample Size)	Syndemic Conditions Evaluated	Adverse Outcomes	Main Results
Chicheto et al., 2023 **[14].**	USA	Quantitative–Cohort	Women with HIV (n = 3282) between 1987 and 2017 from urban and rural areas	Excessive drinking + smoking + depressive symptoms hypertension + diabetes in people with HIV	Mortality	Clustering of two or more conditions affected nearly one in five Women with HIV and was associated with higher mortality.
Costa et al., 2023 **[15].**	Brazil	Quantitative–Cross-sectional	Adults between 18 and 19 years old (n = 2515)	Socioeconomic inequalities + obesity + insulin resistance	Burden of chronic oral diseases (caries and periodontitis)	They found a syndemic framework linking obesity and the Insulin Resistance Phenotype with the Chronic Oral Disease Burden. Socioeconomic Inequalities were associated with a higher Chronic Oral Disease Burden.
Wildman et al., 2022 **[16].**	England	Qualitative–Cross-sectional	Patients with noncommunicable diseases—NCDs (n = 29)	Social circumstances + cardiovascular diseases + diabetes, depression + anxiety during the COVID-19 pandemic	Lower quality of life	The public health response to the pandemic increased the work required to manage conditions in those most vulnerable to harm from COVID-19. Mental distress was amplified by fear of infection and social distancing requirements that removed usual sources of support. Poor housing, low incomes and the need to earn a living further amplified the work of managing daily life and put mental health at risk.
Perelman, 2022 **[17].**	Portugal	Quantitative–Cross-sectional	Adults between 25 and 79 years old (n = 12,052)	Asthma, chronic bronchitis, CVD and cerebrovascular diseases, diabetes, HT, chronic kidney disease (CKD) and obesity	COVID-19 mortality	High socioeconomic inequalities were evident for all eight diseases assessed and were associated with COVID-19 mortality.
Mendenhall, 2022 **[18].**	South Africa	Mixed–Cross-sectional	Adults from neighborhoods of an urban settlement (n = 783)	Stress + multimorbidity (HT + diabetes + hyperlipidemia + chronic pain + HIV)	Lower quality of life	The quality of life impacts of multimorbidity were conditioned by participants’ illness experiences. The strongest finding reveals a robust interaction between a locally designed stress scale and multimorbidity. Stress was associated with medical complications, financial difficulties, family discord and an unsettled future.
Rizk & Shaya, 2022 **[19].**	USA	Quantitative–Ecological	Counties (n = 160)	Burden of NCDs (HT, DM, obesity and COPD) + socioeconomic inequalities measured in four factors (education, employment, poverty and household income)	Lower vaccination rates and worse outcomes from COVID-19	Counties with higher rates of noncommunicable diseases (COPD, obesity, diabetes and hypertension) and socioeconomic disparities had lower COVID-19 vaccination coverage.
Rodríguez et al., 2021 **[20].**	USA	Quantitative–Cross-sectional	Adults with and without HIV (n = 503)	Low education + child abuse + depressive symptoms + HIV status + obesity	Atherosclerotic plaque and elevated blood pressure	A high prevalence of Cardiovascular disease risk measured by carotid atherosclerotic plaque, and elevated systolic and diastolic blood pressure, was identified. There was also a high prevalence of syndemic conditions. Each syndemic condition was associated with increased odds of cardiovascular disease risk, resulting in high risk for detection of atherosclerosis and elevations in blood pressure when multiple syndemic conditions were present.
Chicheto et al., 2021 **[21].**	USA	Quantitative–Longitudinal	Veterans with and without HIV (5621)	Alcohol consumption + smoking + depressive symptoms + HIV status	CVD incidence	Having at least two of the syndemic conditions (smoking, Unhealthy alcohol use, depressive symptoms) was common among veterans. Having two or more of these conditions was associated with increased risk for incident cardiovascular disease, regardless of HIV status, even after adjusting for comorbidities and traditional risk factors.
Cuschieri & Grech, 2021 **[22].**	Malta	Quantitative–Cross-sectional	Adults with a history of COVID-19 (n = 3947)	Multimorbidity (DM + overweight/obesity + CVD dyslipidemia)	Mortality from COVID-19 and years of life lost	Half the study population had a single noncommunicable disease while a third had multimorbidity. Of these, 6.55% were estimated to be at risk of COVID-19 and requiring hospitalization admission. COVID-19 Years of life lost over 12 months was 5228.54 years. The presence of a single chronic disease or multimorbidity from a young age, as identified in this study, is a public health concern.
Chung et al., 2021 **[23].**	Hong Kong	Quantitative–Cross-sectional	Adults with a history of COVID-19 (n = 3074)	Socioeconomic position + multimorbidity (cardiovascular, kidney, nervous system, digestive diseases, diabetes, cancer and HIV)	COVID-19 severity	Despite an independent adverse impact of multimorbidity on COVID-19 severity, it varied across the socioeconomic ladder, with no significant risk among those living in the wealthiest places. Socioeconomic position interacted with multimorbidity to determine COVID-19 severity with a mitigated risk among the socioeconomically advantaged.
Zefp et al., 2020 **[24].**	USA	Quantitative–Cross-sectional	Older men living with HIV who have sex with men (MSM) (n = 281)	Symptoms of depression, symptoms of post-traumatic stress disorder, past physical or sexual abuse, intimate partner violence, stimulant use and excessive alcohol consumption	Worse adherence to treatment in MSM with HIV	The findings suggest that syndemic conditions may impact medication adherence in older MSM living with HIV.
Jones et al., 2020 **[25].**	USA	Quantitative–Cross-sectional	Women with HIV (n = 131)	Low education + obesity + cigarette smoking + depressive symptoms	HIV status, increased blood pressure and inflammation	Syndemic factors may play a role in HIV health status, thereby contributing to an increased risk of transmission. Additionally, the accumulation of syndemic burden may increase the risk of hypertension.
Byg et al., 2016 **[26].**	USA	Quantitative–Cross-sectional	MSM with HIV (n = 88)	Diabetes + HIV	Glycemic control	One-third had inadequate glycemic control, which was correlated with other markers of disease (hypertension and depression) and independently associated with substance use, high triglycerides and unsuppressed HIV viral load.
Mendehall et al., 2015 **[27].**	Kenya	Mixed–Cross-sectional	Patients from a public hospital (n = 100)	Stress + diabetes + infections	Syndemic suffering	Diabetes accompanies a complex web of social, mental and physical suffering among participants. The study indicates that infection intercepts the stress–diabetes interface among low-income residents. Women revealed more social problems, psychological distress and physical morbidities.
Mendenhall & Norris, 2015 **[28].**	USA	Qualitative–Cross-sectional	Black women (n = 27)	Diabetes + HIV	Syndemic suffering	Women conceive syndemic social and health problems as mutually exacerbating, co-constructions of suffering in everyday life. They communicated the factors in their lives that caused stress; they prioritized social problems as opposed to medical ones. They showed how reconstructing families and raising grandchildren after losing children to AIDS was not only socially challenging but also affected how they ate and accepted and managed their diabetes.

HIV: Human Immunodeficiency Virus. NCD: Noncommunicable diseases. MSM: Men who have sex with men.

**Table 2:** Description of the application of the key elements of syndemic theory.

Studies	Measurement Level		Key Elements			Classification	
	Population	Individual	Concentration	Bio–Social Interaction	Bio–Bio Interaction	Syndemic ^1^	Syndemic Potential ^2^
Chicheto et al., 2023 **[14].**	No	Yes	No	Yes	No	-	✓
Costa et al., 2023 **[15].**	Yes	Yes	Yes	Yes	Yes	✓	-
Wildman et al., 2022 **[16].**	No	Yes	No	Yes	No	-	✓
Perelman, 2022 **[17].**	No	Yes	Yes	No	No	-	✓
Mendenhall et al., 2022 **[18].**	Yes	Yes	Yes	Yes	Yes	✓	-
Rizk & Shaya, 2022 **[19].**	Yes	No	Yes	Yes	No	-	✓
Rodríguez et al., 2021 **[20].**	No	Yes	No	Yes	No	-	✓
Chicheto et al., 2021 **[21].**	No	Yes	No	Yes	No	-	✓
Cuschieri & Grech, 2021 **[22].**	Yes	Yes	No	No	No	-	-
Chung et al., 2021 **[23].**	Yes	Yes	Yes	Yes	No	-	✓
Zefp et al., 2020 **[24].**	No	Yes	No	Yes	No	-	✓
Jones et al., 2020 **[25].**	No	Yes	No	Yes	Yes	-	✓
Byg et al., 2016 **[26].**	No	Yes	Yes	Yes	No	-	✓
Mendehall et al., 2015 **[27].**	Yes	Yes	Yes	Yes	Yes	✓	-
Mendehall et al., 2015 **[28].**	No	Yes	Yes	Yes	No	-	✓

^1^ Syndemics: Studies with interactions that involve the clustering of at least two diseases or conditions within a specific population. Bio–bio interactions of these health burdens result in worse health outcomes and evidence the influence of shared social factors within populations that further exacerbate biological conditions creating a true synergy rather than simply co-occurrence of disease or the addition of various social and health factors [7,8]. ^2^ Potential syndemics: studies that do not fully develop the bio–bio and bio–social interaction.

## Data Availability

The raw data supporting the conclusions of this article will be made available by the authors on request.

## Supplementary Materials

The following supporting information can be downloaded at: https://www.mdpi.com/article/10.3390/tropicalmed9090196/s1, Table S1: Syndemics between cardiometabolic and infectious diseases in adults; Table S2: Specific searches.
