# Supplementary material for: Convergence between Cardiometabolic and Infectious Diseases in Adults from a Syndemic Perspective: A Scoping Review
Source: Trop Med Infect Dis. 2024 Aug 26;9(9):196. doi: 10.3390/tropicalmed9090196 (PMC11435530; doi:10.3390/tropicalmed9090196)
Supplement: Supplementary file 1 [file tropicalmed-09-00196-s001.zip › tropicalmed-3121678-supplementary.pdf]

Supplementary Table S1. Syndemics between cardiometabolic and infectious diseases in adults

Database	Términos MESH/Key words	Entry terms
Pubmed	1.**Syndemic:** Synergistically-interacting health conditions producing excess disease occurrence in a population.<br>Year introduced: 2019	Syndemics
	2. **coexistent conditions:** Used with diseases to indicate conditions that co-exist or follow, i.e., co-existing diseases, complications, or sequelae. Year introduced: 1966	associated disease<br>coexistent disease<br>concomitant disease<br>concomitant conditions<br>sequelae
	3. **Convergence diseases:** No es termino MESH	
	4. **Cardiometabolic Syndromes: A cluster of** symptoms that are risk factors for cardiovascular diseases and type 2 diabetes mellitus. the major components of metabolic syndrome include abdominal obesity; atherogenic dyslipidemia; hypertension; hyperglycemia; insulin resistance; a proinflammatory state; and a prothrombotic (thrombosis) state. year introduced: 2018 (2002)	Metabolic Syndromes<br>Metabolic Cardiovascular Syndrome<br>Cardiovascular Syndrome, Metabolic<br>Metabolic Syndrome X<br>Insulin Resistance Syndrome X<br>Syndrome X, Insulin Resistance
	5. **Cardiovascular Diseases:** pathological conditions involving the cardiovascular system including the heart; the blood vessels; or the pericardium.	Cardiovascular Disease<br>Disease, Cardiovascular<br>Diseases, Cardiovascular
	6. **Noncommunicable Diseases:** Diseases which are typically non-infectious in origin and do not transmit from an affected individual to others. The four main types of noncommunicable diseases are CARDIOVASCULAR DISEASES (e.g., heart attacks and stroke), CANCER, chronic respiratory diseases (e.g., chronic obstructive pulmonary disease and asthma) and diabetes mellitus.<br><br>Year introduced: 2018	Non-infectious Diseases<br>Non-communicable Diseases
	7**. Communicable Diseases**<br>An illness caused by an infectious agent or its toxins that occurs through the direct or indirect transmission of the infectious agent or its products from an infected individual or via an animal, vector or the inanimate environment to a susceptible animal or human host.	Infectious Diseases<br>Disease, Communicable<br>Diseases, Communicable
	8. **Infections:** Invasion of the host organism by microorganisms or their toxins or by parasites that	Infection and Infestation<br>Infestation and Infection

	can cause pathological conditions or diseases. Year introduced: 2020	Infections and Infestations<br>Infestations and Infections<br>Infection

Supplementary Table S2. Specific searches

N°	Searches	Result
1	(Syndemic[Title/Abstract]) OR (Syndemics[Title/Abstract])	736
2	((((coexistent conditions[Title/Abstract]) OR (associated disease[Title/Abstract])) OR (coexistent disease[Title/Abstract])) OR (concomitant disease[Title/Abstract])) OR (concomitant conditions[Title/Abstract])	6.854
3	Convergence diseases[Title/Abstract]	3977
4	**1 OR 2 OR 3**<br>(((Syndemic[Title/Abstract]) OR (Syndemics[Title/Abstract])) OR (((((coexistent conditions[Title/Abstract]) OR (associated disease[Title/Abstract])) OR (coexistent disease[Title/Abstract])) OR (concomitant disease[Title/Abstract])) OR (concomitant conditions[Title/Abstract]))) OR (Convergence diseases[Title/Abstract])	11.556
5	((((((Cardiometabolic Syndromes) OR (Metabolic Syndromes)) OR (Metabolic Cardiovascular Syndrome)) OR (Cardiovascular Syndrome, Metabolic)) OR (Metabolic Syndrome X)) OR (Insulin Resistance Syndrome X)) OR (Syndrome X, Insulin Resistance)	93.648
6	(((Cardiovascular Diseases) OR (Cardiovascular Disease)) OR (Disease, Cardiovascular)) OR (Diseases, Cardiovascular)	2.657.517
7	((Noncommunicable Diseases) OR (Non-infectious Diseases)) OR (Non-communicable Diseases)	60.688
8	**5 OR 6 OR 7**<br>((((((((Cardiometabolic Syndromes) OR (Metabolic Syndromes)) OR (Metabolic Cardiovascular Syndrome)) OR (Cardiovascular Syndrome, Metabolic)) OR (Metabolic Syndrome X)) OR (Insulin Resistance Syndrome X)) OR (Syndrome X, Insulin Resistance)) OR ((((Cardiovascular Diseases) OR (Cardiovascular Disease)) OR (Disease, Cardiovascular)) OR (Diseases, Cardiovascular))) OR (((Noncommunicable Diseases) OR (Non-infectious Diseases)) OR (Non-communicable Diseases))	2.772.849
9	(((Communicable Diseases) OR (Infectious Diseases)) OR (Disease, Communicable)) OR (Diseases, Communicable)	843.077
10	(((((Infections) OR (Infection and Infestation)) OR (Infestation and Infection)) OR (Infections and Infestations)) OR (Infestations and Infections)) OR (Infection)	3.613.321
11	**9 OR 10**<br>((((Communicable Diseases) OR (Infectious Diseases)) OR (Disease, Communicable)) OR (Diseases, Communicable)) OR ((((((Infections) OR (Infection and Infestation)) OR (Infestation and Infection)) OR (Infections and Infestations)) OR (Infestations and Infections)) OR (Infection))	3.722.965

12	**8 AND 11**<br><br>((((((((Cardiometabolic Syndromes) OR (Metabolic Syndromes)) OR (Metabolic Cardiovascular Syndrome)) OR (Cardiovascular Syndrome, Metabolic)) OR (Metabolic Syndrome X)) OR (Insulin Resistance Syndrome X)) OR (Syndrome X, Insulin Resistance)) OR ((((Cardiovascular Diseases) OR (Cardiovascular Disease)) OR (Disease, Cardiovascular)) OR (Diseases, Cardiovascular))) OR (((Noncommunicable Diseases) OR (Non-infectious Diseases)) OR (Non-communicable Diseases))) AND (((((Communicable Diseases) OR (Infectious Diseases)) OR (Disease, Communicable)) OR (Diseases, Communicable)) OR ((((((Infections) OR (Infection and Infestation)) OR (Infestation and Infection)) OR (Infections and Infestations)) OR (Infestations and Infections)) OR (Infection)))	248.663
13	**4 AND 12**<br><br>((((Syndemic[Title/Abstract]) OR (Syndemics[Title/Abstract])) OR (((((coexistent conditions[Title/Abstract]) OR (associated disease[Title/Abstract])) OR (coexistent disease[Title/Abstract])) OR (concomitant disease[Title/Abstract])) OR (concomitant conditions[Title/Abstract]))) OR (Convergence diseases[Title/Abstract])) AND (((((((((Cardiometabolic Syndromes) OR (Metabolic Syndromes)) OR (Metabolic Cardiovascular Syndrome)) OR (Cardiovascular Syndrome, Metabolic)) OR (Metabolic Syndrome X)) OR (Insulin Resistance Syndrome X)) OR (Syndrome X, Insulin Resistance)) OR ((((Cardiovascular Diseases) OR (Cardiovascular Disease)) OR (Disease, Cardiovascular)) OR (Diseases, Cardiovascular))) OR (((Noncommunicable Diseases) OR (Non-infectious Diseases)) OR (Non-communicable Diseases))) AND (((((Communicable Diseases) OR (Infectious Diseases)) OR (Disease, Communicable)) OR (Diseases, Communicable)) OR ((((((Infections) OR (Infection and Infestation)) OR (Infestation and Infection)) OR (Infections and Infestations)) OR (Infestations and Infections)) OR (Infection))))	374
14	**humanos y Not cáncer filter**<br><br>(((((Syndemic[Title/Abstract]) OR (Syndemics[Title/Abstract])) OR (((((coexistent conditions[Title/Abstract]) OR (associated disease[Title/Abstract])) OR (coexistent disease[Title/Abstract])) OR (concomitant disease[Title/Abstract])) OR (concomitant conditions[Title/Abstract]))) OR (Convergence diseases[Title/Abstract])) AND (((((((((Cardiometabolic Syndromes) OR (Metabolic Syndromes)) OR (Metabolic Cardiovascular Syndrome)) OR (Cardiovascular Syndrome, Metabolic)) OR (Metabolic Syndrome X)) OR (Insulin Resistance Syndrome X)) OR (Syndrome X, Insulin Resistance)) OR ((((Cardiovascular Diseases) OR (Cardiovascular Disease)) OR (Disease, Cardiovascular)) OR (Diseases, Cardiovascular))) OR (((Noncommunicable Diseases) OR (Non-infectious Diseases)) OR (Non-communicable Diseases)))	**281**

	AND (((((Communicable Diseases) OR (Infectious Diseases)) OR (Disease, Communicable)) OR (Diseases, Communicable)) OR ((((((Infections) OR (Infection and Infestation)) OR (Infestation and Infection)) OR (Infections and Infestations)) OR (Infestations and Infections)) OR (Infection)))) AND (humans[Filter])) NOT (cancer[Title] AND (humans[Filter])) Filters: Humans	
